# Computational method allowing Hydrogen-Deuterium Exchange Mass Spectrometry at single amide Resolution

**DOI:** 10.1038/s41598-017-03922-3

**Published:** 2017-06-19

**Authors:** Chris Gessner, Wieland Steinchen, Sabrina Bédard, John J. Skinner, Virgil L. Woods, Thomas J. Walsh, Gert Bange, Dionysios P. Pantazatos

**Affiliations:** 1 Indiana University, Department of Informatics and Computing, Bloomington, IN USA; 2 Weill Cornell Medicine, Transplantation-Oncology Infectious Disease Program, Division of Infectious Diseases, 1300 York Ave, New York, NY 10065 USA; 3 GlaxoSmithKline, Platform Technology & Science, Collegeville Road, Collegeville, Pennsylvania 19426 United States; 4 iHuman Institute, ShanghaiTech University, 99 Haike Road, Pudong, Shanghai China; 5 Philipps-University Marburg, Faculty of Chemistry & LOEWE Center for Synthetic Microbiology Hans-Meerwein-Strasse, 35043 Marburg, Germany

## Abstract

Hydrogen-deuterium exchange (HDX) coupled with mass spectrometry (HDXMS) is a rapid and effective method for localizing and determining protein stability and dynamics. Localization is routinely limited to a peptide resolution of 5 to 20 amino acid residues. HDXMS data can contain information beyond that needed for defining protein stability at single amide resolution. Here we present a method for extracting this information from an HDX dataset to generate a HDXMS protein stability fingerprint. High resolution (HR)-HDXMS was applied to the analysis of a model protein of a spectrin tandem repeat that exemplified an intuitive stability profile based on the linkage of two triple helical repeats connected by a helical linker. The fingerprint recapitulated expected stability maximums and minimums with interesting structural features that corroborate proposed mechanisms of spectrin flexibility and elasticity. HR-HDXMS provides the unprecedented ability to accurately assess protein stability at the resolution of a single amino acid. The determination of HDX stability fingerprints may be broadly applicable in many applications for understanding protein structure and function as well as protein ligand interactions.

## Introduction

Rapid characterization of protein conformation, dynamics, and mapping of protein-ligand interface sites is increasingly being performed by employing hydrogen-deuterium exchange mass spectrometry (HDXMS)^1^. This methodology is advantageous in terms of sensitivity, speed and capability of analyzing large proteins and protein-ligand complexes^2^. HDX is pH- and temperature-dependent^3, 4^ and involves the reversible exchange of backbone amide hydrogens in a protein with deuterium (D) from the solvent. HDX rates of proteins are determined by labeling of backbone amides with deuterium oxide (D_2_O) and are measured by liquid chromatography mass spectrometry (LCMS). In order for HDX to occur, the amide hydrogen must physically contact the D from the solvent through a local or global unfolding mechanism that may necessitate disruption of any hydrogen bonding.

The relationship of HDX and protein structure dynamics has been well-characterized^5, 6^. It is an invaluable method for interrogating protein structure fluctuations and representing localized dynamics^6^. The HDX rate of protein amides is greatly impacted by their solvent accessibility in the context of the protein structure and may vary as many as 9 orders of magnitude on the time scale^7–10^. This wide range of HDX rates represents enormous differences in the protein stability spectrum and might also be expressed as the free energy of exchange (ΔG_ex_)^8^. Consequently, the stability fingerprint of a protein structure may then also be defined as the ΔG_ex_ versus the number of amino acids.

The ultimate advantages of HDXMS in many areas of life sciences^10–15^ has led to a rapid expansion of this technology^1^ and fostered the development of software packages for high-throughput analysis^16, 17^. The HDXMS workflow has been thoroughly described in numerous applications^17–21^. Briefly, proteins or their complexes are incubated in deuterated buffer and after defined time points the reaction is quenched by acidic quench buffer acids (pH 2.7). Subsequently, samples are digested proteolytically to generate peptide fragments^17^. Localization and quantification of D-incorporation into each peptide is performed by LCMS with resulting data analyzed using specialized software^18^. Ultimately, the HDXMS data are then either projected as the percentage of D-incorporation (%D) onto the amino acid sequence (ribbon heat maps) or onto a protein structure (3D heat maps)^19, 22^. At present, HDXMS analysis is mainly restricted to peptide resolution governed by the length of the obtained peptides. Analysis of HDXMS data at single amino acid resolution would lead to a much greater insight and understanding of protein structure, dynamics and function. However, software tools enabling this task have yet to be (further) developed.

In this study, we present a software tool enabling HDXMS analysis at highest resolution (HR-HDXMS) by determining single amide HDX rates from acquired HDXMS centroid data. HR-HDXMS utilizes D-incorporation from multiple overlapping peptide fragments and multiple D-labeling time points to determine single amide HDX rates in proteins. These can be collectively presented as the HDX stability fingerprint of a given protein. To demonstrate the feasibility of our approach, we used the structurally and dynamically well-characterized α-spectrin variant as a model protein^23^. Our software improved the spatial resolution of protein dynamics by providing a superior visualization of the spatial distribution of the stability profile of the structure. Our application demonstrates the utility of HR-HDXMS and HDX stability profiles for evaluating and understanding protein structure and function.

## Results

### Conventional HDXMS analysis of spectrin

**Figure 1:**
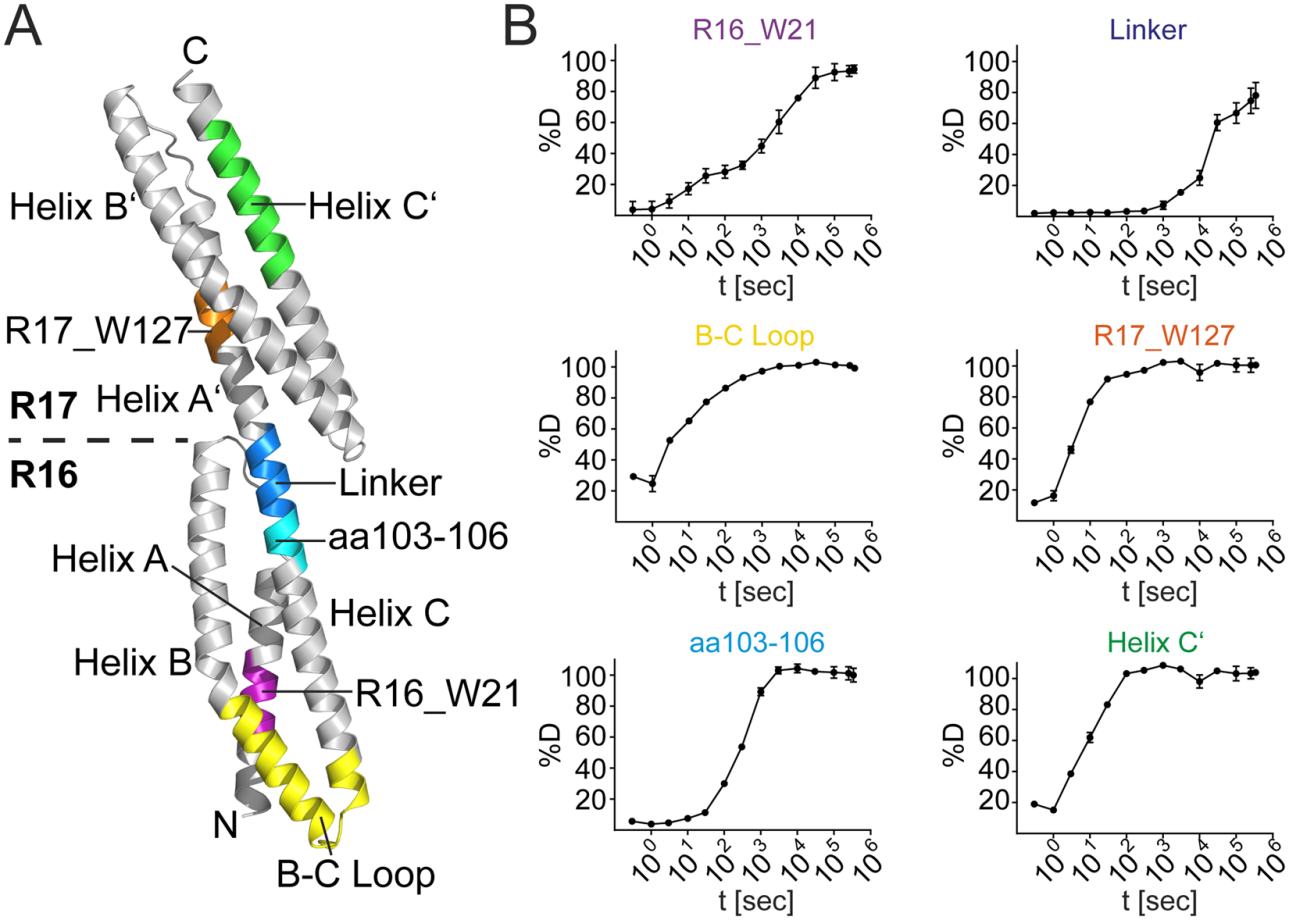
Conformational dynamics of R1617. (**A**) The crystal structure of repeats 16 & 17 of α-spectrin (PDB: 1CUN^23^) is shown in cartoon representation. Regions shown in (**B**) are colored and indicated. (**B**) Deuterium uptake of regions of R1617: R16_W21 (aa 15–21), B-C Loop (aa 61–84), aa 103–106, Linker (aa 108–114), R17_W127 (aa 125–128) and Helix C′ (aa 200–213).

For the development of HR-HDXMS we chose a structurally well-characterized variant of α-spectrin from chicken brain containing repeats 16 and 17 (further called R1617)^23^. R1617 is composed of two triple-helical bundles joined by a 5 amino acid linker with α-helical features^23^ (Fig. 1A). In order to generate a sufficient amount of overlapping peptides covering the complete amino acid sequence of spectrin, we employed a combination of the proteases pepsin and protease type XIII from *Aspergillus saitoi* (FPXIII) during LCMS analysis. We obtained approximately 200 peptides ranging from 4 to 41 amino acids and covering the entire amino acid sequence of spectrin (Fig. S1 and Table S1). Each peptide was manually reviewed for the correct assignment of charge state and the quality of the spectrum. The combined use of pepsin and FPXIII rather than each alone produced many small peptides with high overlap (Fig. S1). Moreover, this approach significantly increased the redundancy in coverage per amino acid in most regions greater than 2-fold and in some regions even greater than 5-fold. (Fig. S1). Furthermore, we were able to obtain many peptides with charge states ranging from +1 to +3. Multiple charge states are beneficial to estimate the degree of D-incorporation more accurately because the mass of the peptide should be the same at each charge state.

The percentage of D-incorporation over time for each peptide depends on the localization of the peptide within the protein structure. The use of 13 time points for the measurement of %D provided complete and accurate information about which regions of R1617 were less or more accessible to HDX and how fast the exchange occurred (Fig. 1A and B). We classified different regions of R1617 based on their velocity of HDX by determining the time required for 50% D-incorporation to occur on a peptide (Table S1). With this approach, peptides with 50% D-incorporation <1000 seconds were classified as fast exchanging (135 peptides), those with 50% D-incorporation between 1000–3000 seconds were intermediate exchanging (36 peptides), and peptides that reached 50% D-incorporation above 3000 seconds as slow exchanging (31 peptides). Examples for the fast exchange were the B-C Loop (<3 seconds), Helix C′ (<10 seconds) and a peptide in repeat 17 spanning W127 (R17_W127) residing in helix A′ (<10 seconds, Fig. 1B). Also, amino acids 103–106 preceding the linker region of spectrin incorporate over 50%D after just 300 seconds, while the slow exchanging linker (*i.e*. amino acids 108–114) requires 100-times longer to reach similar deuterium incorporation (Fig. 1B). A peptide (R16_W21) in repeat 16 that includes W21 residing in Helix A of R1617 displays an intermediate D-incorporation. However, two distinct kinetics of deuterium uptake were apparent from the time course with a rather fast and slow exchanging species being overlaid (Fig. 1B). Our data confirm that R1617 is a highly dynamic molecule. Nevertheless, with the conventional way of analyzing HDXMS data subtle differences within the dynamic behavior of spectrin cannot be identified or be attributed to single amino acid residues.

### Theoretical concept of HR-HDXMS

**Figure 2:**
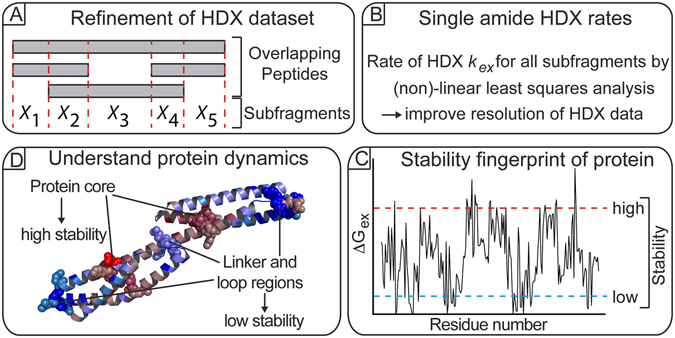
Workflow of HR-HDXMS. (**A**) Amino acid subfragments are determined from overlapping peptides generated by proteolytic digest of the protein. (**B**) The rate of hydrogen-deuterium exchange (*k*_*ex*_) of each subfragment is calculated by linear and non-linear least squares analysis. (**C**) The free energy value ΔG_ex_ derived from *k*_*ex*_ is used to generate a stability fingerprint of the protein. (**D**) The protein fingerprint helps in understanding protein dynamics and allows predictions about residues that might be essential for protein stability and/or function.

To improve the resolution in visualizing HDXMS data, we aimed to develop a computational algorithm to determine the hydrogen-deuterium exchange rates and the thermodynamic stability of single amides from an HDXMS dataset of a protein (Fig. 2). Central to this approach is the generation of a peptide library containing multiple overlapping peptides that are produced by combined proteolysis of the protein using pepsin and FPXIII followed by the determination of subfragments between all peptides. In this a subfragment *X* is defined as the smallest common overlapping sequence between two given peptides (Fig. 2A). Subsequently, the amount of deuterium incorporated on each subfragment *X* is computationally determined by a linear least squares minimization of a global error (GE) function (Fig. 2B and Fig. S2). Deuterium shifts for each subfragment are then determined from the difference of D-incorporation of the subfragment between each successive labeling time point. Next, the HDX rates (*k*_*ex*_) for each subfragment are estimated by fitting the exponent in equation 1 using the D-shifts of the calculated subfragments (Fig. 2B and Fig. S3). Consequently, *k*_*ex*_ for each *X* represents an average of the exchange rates from all amides within *X*. The exchange rates from the subfragments are used to seed initial starting rates for the non-linear least squares optimization function that solves for the *k*_*ex*_ of all amides (Fig. 2B and Fig. S3). The so-obtained exchange rates *k*_*ex*_ of each amide can then be converted into the free energy of hydrogen-deuterium exchange (ΔG_ex_) according to the Gibbs free energy equation allowing for a precise understanding of protein dynamics on the single amino acid level (Fig. 2C and D). Taken together, this procedure should enable the determination of *k*_*ex*_ values of single amides from HDXMS datasets containing a sufficiently large number of overlapping peptides with redundant sequence coverage.

### Subfragmentation improves the spatial resolution of HDXMS

To learn more about subtle changes within the dynamics of R1617 that might not be visible by conventional HDXMS evaluation, we applied our HR-HDXMS software to the HDXMS dataset. Subfragments were determined from overlapping peptides followed by determination of the deuterium incorporation and the rate of hydrogen-deuterium exchange *k*_*ex*_ of each subfragment by linear and non-linear least squares analysis (see above). These steps drastically improved the spatial resolution of the dynamics of R1617 (Fig. S4). In those areas of R1617 where many overlapping peptide fragments were available, deuterium uptake could even be determined for single amino acids (compare to Figs S1 and S4).

**Figure 3:**
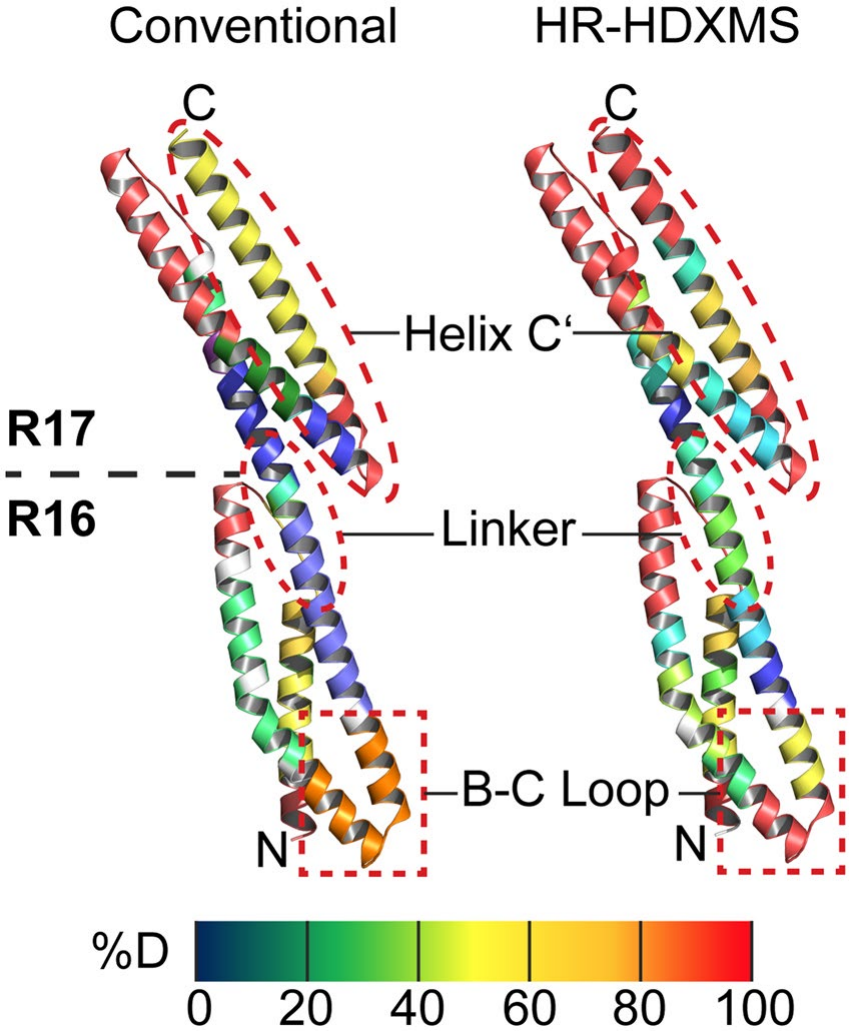
Improved resolution of R1617 conformational dynamics by HR-HDXMS. Deuterium uptake of R1617 mapped onto its crystal structure (PDB: 1CUN^23^). Data analysis by HR-HDXMS (*right*) provides a superior spatial resolution of deuterium uptake compared to ‘conventional’ HDX data analysis (*left*).

To visually inspect the improvements in resolution, we projected the results of ‘conventional’ HDXMS (see above) and HR-HDXMS data analysis on the crystal structure of R1617 (Fig. 3). Helix C′ could now be subdivided into four segments with different dynamic properties rather than two seen with the conventional approach (from the N- to C-terminus of helix C′: >90, 50, 20 and >90% D) (Fig. 3). Another example was found for the dynamics of the B-C Loop. While the conventional approach displayed intermediate-high dynamics (70% D), the HR-HDXMS heat map was further subdivided into three categories of dynamics including regions of very high (>90% D) over intermediate (50% D) to low (20% D) dynamics from the N- to the C-terminus of the B-C Loop (Fig. 3). Moreover, within the linker region the number of segments exhibiting different dynamics could be increased approximately two-fold allowing for a more detailed understanding (see below). Taken together, our approach of generating a subfragment map clearly refines analysis of HDXMS as exemplified by R1617 (Fig. 3).

### HR-HDXMS allows stability fingerprints at single amino acid residue resolution

**Figure 4:**
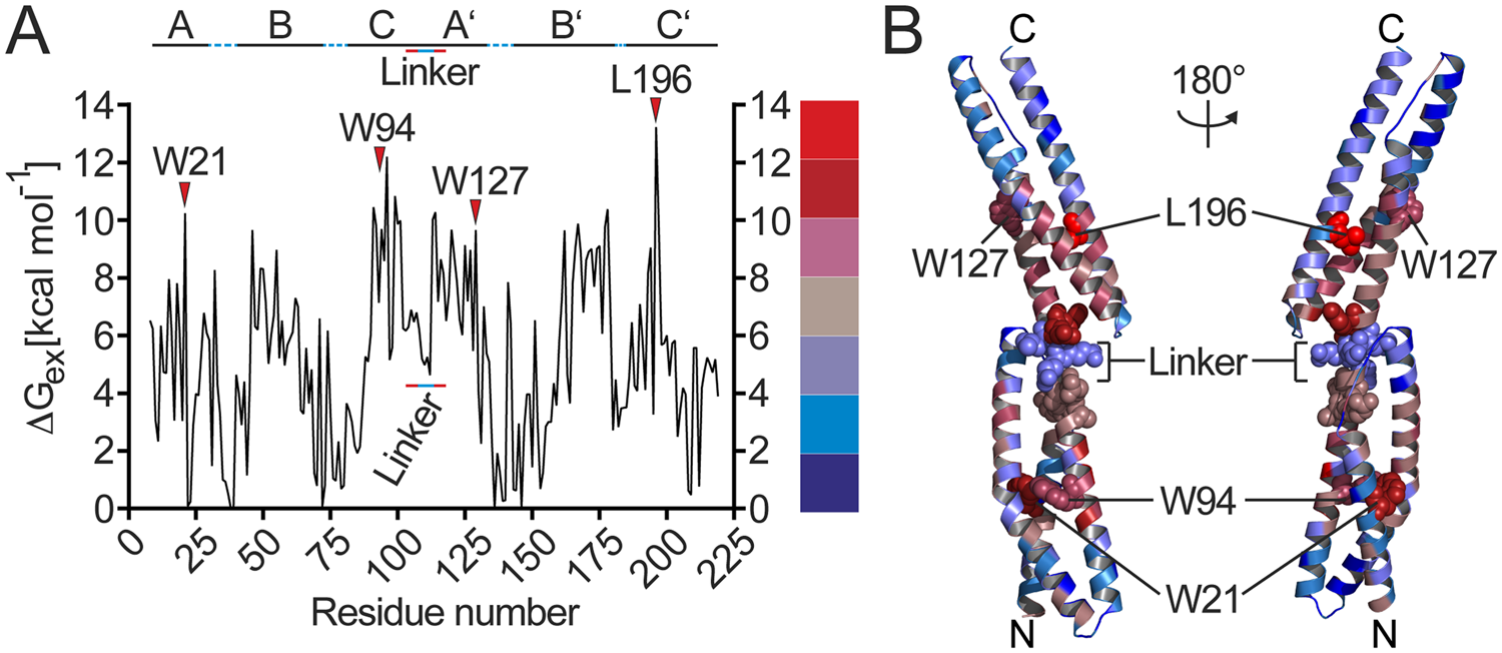
Stability fingerprint of R1617 determined by HR-HDXMS. (**A**) Plot of the free energy value ΔG_ex_ for each amino acid residue of R1617. The linker region between repeats 16 and 17 and amino acids adjacent to the linker are indicated by solid blue and red lines, respectively. Amino acid residues exhibiting high stability are indicated. (**B**) The crystal structure of R1617 (PDB: 1CUN^23^) colored according to the ΔG_ex_ values shown in (**A**).

Following our theoretical considerations (see above), we wanted to take our analysis further, eventually to determine stability fingerprints at the single amide level. Therefore, the exchange rates from each subfragment were used to seed initial starting rates for the nonlinear least squares function solving for the exchange rate of each amide (*k*_*ex*_) (equation 7). The *k*_*ex*_ was converted into the free energy of exchange (ΔG_ex_) according to the Gibbs Free Energy equation (ΔG_exchange,i_ = −RT ln(*k*_*ex,i*_/k_int,i_)) where *k*_*ex,i*_ and *k*_*int,i*_ are the experimental and intrinsic (random coil) exchange rates, respectively, at an amide i as determined from the intrinsic rates of random coil model peptides^24, 25^. These ΔG_ex_ for each amide were plotted versus the position of the amino acid residue (Fig. 4A). The ΔG_ex_ values range from 0 kcal/mol (*i.e*. prolines lacking the amide hydrogen) to 14 kcal/mol. Generally, lower and higher ΔG_ex_ values correlate to faster and slower HDX rates of the amide, respectively^6, 7, 26^. Therefore, lower ΔG_ex_ values represent amides of amino acid residues that are more dynamic and, *vice versa*, higher ΔG_ex_ represent amides of residues that are less dynamic.

The HDX stability fingerprint of R1617 as determined by our HR-HDXMS approach displays a jagged profile with an observable pattern of stability with a large window of variation in amide dynamics (Fig. 4A). Generally, higher ΔG_ex_ values are observed around the centers of the α-helices and lower ΔG_ex_ values are observed in the loop regions connecting the α-helices. Noteworthy, the amides of four amino acids (*i.e*. amino acids 109–112) constituting the linker that connects the repeats 16 and 17 exhibit a local minimum in ΔG_ex_ while residues preceding and following the linker showed much higher ΔG_ex_ values (Fig. 4A). The functional consequences of this keen discrimination between the linker region and the C- and N-terminal α-helices of the spectrin repeats 16 and 17 are discussed below.

To challenge the validity of our approach, we examined the accuracy upon which the HDX stability fingerprint could correctly identify amino acids or locations on the structure of R1617 that have previously been shown to confer stability or impose flexibility^23, 27, 28^. For this, the ΔG_ex_ values were projected onto the crystal structure of R1617 resulting in a superior spatial resolution of dynamics when compared to our previous subfragment analysis (Fig. 4B, and compare with Fig. 3). Our analysis revealed the amides of three tryptophan residues (*i.e*. W21, W94 within repeat 16 and W127 in repeat 17) and leucine at position 196 to exhibit very high ΔG_ex_ values. These residues localize in the center of the triple helical bundles forming repeat 16 and 17 (ref. 23). Notably, W21 and W94 have already been shown to significantly confer stability of the triple helical core^27, 28^, consolidating the validity of our HR-HDXMS approach to determine a stability fingerprint.

Taken together, these results clearly demonstrate that our HR-HDXMS approach allows unraveling the contribution of single amino acids to the overall dynamics of protein structures exemplified by spectrin.

### HR-HDXMS agrees with single amide HDX rates obtained by NMR

Traditionally, single amide HDX rates have been determined by nuclear magnetic resonance (NMR)^29, 30^. Therefore, we extended the validation of our HR-HDXMS method against data obtained by NMR. To do so, we performed HR-HDXMS on a dataset obtained for *Staphylococcus* nuclease (SNase) comprising 341 peptides and 10 sampled time points^31^. In the initial determination of deuterium incorporation by linear least squares analysis (equation 1), a large number of residues exhibited a high residual error (Fig. S5A). To circumvent this problem that might be caused by poor data quality or incorrect peptide assignment, we eliminated peptides with an error ≥2 deuterium. After exclusion of those peptides, the residual error for each peptide from the linear optimization (equation 2) could be reduced below 1 deuterium for the remaining 206 filtered fragments (Fig. S5B). Although approximately 40% of peptides are lost during this procedure reducing the obtainable resolution, the quality of deuterium incorporation assignment for the remaining peptides is significantly raised.

For comparison of the stability fingerprints obtained by HR-HDXMS and NMR, the analogous NMR exchange rate data of SNase served as a gold standard^9, 26^. At first, the *k*_*ex*_ rates obtained by NMR were used to determine the stability fingerprint of SNase by the HR-HDXMS algorithm. The ΔG_ex_ values obtained by global error minimization applying HR-HDXMS are almost identical to the ΔG_ex_ values obtained from the experimental data (Fig. S6A). Also, the stability fingerprint of SNase obtained by determination of *k*_*ex*_ values from HDXMS dataset resulted in a good overlay with the NMR-derived stability fingerprint (Fig. S6B). These results further demonstrate the feasibility of HR-HDXMS even when suboptimal HDX data are available (see above) and show that the accuracy of the fingerprint relies on the quality of the seeding rates in the nonlinear solver.

## Discussion

### HR-HDXMS as tool to refine HDX data to single amide resolution

In this work, we present HR-HDXMS as suitable tool for refining HDXMS data to single amide resolution. Therefore, HR-HDXMS opens a new route to delineate the individual contribution of single amino acids to the overall dynamics of proteins and their macromolecular assemblies. To this end, suitability of HR-HDXMS was tested with repeats 16 & 17 of chicken brain α-spectrin (R1617), an enormously well characterized protein in terms of structure^23, 32^, biophysical characteristics^27, 33^, and postulated mechanisms of function. As exemplified, HR-HDXMS was able to recapitulate all known features of spectrin structure and dynamics, but much more provided further insights into the dynamic features of spectrin (see below). However, whether these are of functional relevance remains to be further investigated.

The HR-HDXMS approach involves a simple two-phase numerical technique. A linear least squares fit is performed for the initial estimation of amide exchange rates from overlapping peptide fragments followed by a non-linear least squares fit solving for individual amide exchange rates within each peptide. We generated an HDXMS dataset of repeats 16 & 17 of chicken brain α- spectrin, which provided 2600 overlapping constraints that were a product of 200 peptides and 13 time points of deuterium labeling for performing the linear and non-linear fits. Our study showed that successful application of HR-HDXMS critically relies on: the generation of multiple overlapping peptide fragments, general data quality, correction for D-loss into the solvent by incorporating residue-specific back-exchange rates in the non-linear fit, and imposing appropriate boundaries for HDX rates during non-linear optimization to serve as constraints for the fit. We believe that the generation of multiple overlapping peptides (allowing the subfragment assignment) is most critical for the success of HR-HDXMS. This issue can be addressed by the use of the two proteases pepsin and FPXIII. Overall, HR-HDXMS seems to offer a promising way to analyze HDXMS datasets at single amide resolution. However, further studies are required to challenge the suitability of our approach especially with respect to larger proteins and macromolecular assemblies where the amount of overlapping peptides and redundant sequence coverage becomes a greater challenge.

### Advantages of HR-HDXMS over other methods and alternatives

Traditionally, single amide HDX rates have been determined by nuclear magnetic resonance (NMR)^29, 30^. While NMR is powerful to accurately measure single amide HDX rates, it is usually limited in protein size (<30 kDa molecular weight). A great advantage of HDXMS is its ability to analyze proteins and macromolecular assemblies extending molecular weights over 100 kDa. Moreover, our study shows that HR-HDXMS is able to recapitulate single amide HDX rates obtained by NMR as exemplified by SNase.

Mass spectrometry is also capable of determining the D-incorporation at a single amide through stepwise peptide fragmentation employing electron capture dissociation (ECD) and electron transfer dissociation (ETD)^34–37^. Collision-induced dissociation (CID) fragmentation approaches have commonly been used to improve coverage and sublocalization of D-labeling. However, this approach typically suffers from deuterium scrambling^38^ among adjacent amides making reliable sublocalization challenging. HR-HDXMS alleviates the drawbacks of CID, because it relies on the total D-incorporation on the peptide irrespective of any scrambling occurring during CID fragmentation.

Fragmentation methods such as ECD^39^ and ETD^36, 40^ also allow for determination of single amide D-incorporation by inducing stepwise fragmentation. However, the conventional use of these methods to determine D-incorporation at the single amide level is limited. ETD requires peptides with higher charge states to work effectively^41^. Therefore, not all peptides will be amenable to ETD fragmentation.

Other mathematical and analytical approaches have recently been developed for determining single amide rates and/or improving the resolution in HDXMS datasets^31, 42–46^. These approaches include a mathematical model for predicting the assignment of HDX rate classes to amino acid segments from overlapping peptides^46^, a statistical approach to quantify the difference of HDX between two states^42^, residue averaging of the mid-point values of HDX^43^, and utilization of the shape of the isotopic envelope to determine the distribution of deuterium on the amides of the peptide by the software HDSite^31^. HDSite demonstrates the feasibility of determining exchange rates at single amide resolution, but it functions optimally only with high-resolution data providing enough information to produce a well-defined isotopic envelope. Implementation of HDSite involves a separate iteration for each peptide to determine the amide D-occupancy in the peptide and a separate fit to an exponential to determine the HDX rate for each residue based on the previously determined amount of D on the amide at each time point. In contrast to HDSite, HR-HDXMS performs a single global linear optimization iteration to simultaneously determine the D level on all subfragments from overlapping peptides and then a single global non-linear iteration using a non-linear optimizer to simultaneously determine all HDX rates for all amides. One drawback of the HR-HDXMS software is that it assumes all HDX to be governed by EX2 kinetics, and in its present version does not take EX1 kinetics into account.

Taken together, both HDSite and HR-HDXMS depend on a sufficiently high number of overlapping fragments, number of time points, length of the sequence, and number of replicates. These issues represent the most challenging limitation of both approaches. Nevertheless, our validation of HR-HDXMS demonstrated its robustness to correctly calculate the stability profile of a protein.

### Lessons learned on the dynamics of spectrin by HR-HDXMS

Our HR-HDXMS approach improved the spatial resolution of the dynamics of the α-spectrin tandem repeat (R1617) to the single amide level. Based on the crystal structure of R1617, two models of elasticity and flexibility were proposed^23^. The first model described the flexibility imposed by the linker^23, 47^ through helix bending at the linker region. The second one suggested high flexibilities along the α-helices and their connecting loops due to their conformational rearrangement^18^. Interestingly, our HR-HDXMS analysis supports both models and extends them further. We found substantial stability gradients at the center of the α-helices that gradually decrease towards the helical ends (Fig. 4B). Probably, gradients of stability near the helical ends may facilitate the structural transition from helical propensity into loop geometry. With respect to the linker, conventional analysis of our HDXMS supports the highly dynamic behavior of the linker as already inferred from the crystal structure (Fig. 3). However, the refinement in spatial resolution provided by our HR-HDXMS approach revealed that residues 104–106 preceding the linker exhibited higher ΔG_ex_ than the linker residues 108–112 that exhibited a local minimum in ΔG_ex_. Thereby, we could identify specific amino acids that possibly confer less or more stability within the linker region. Therefore, our HR-HDXMS analysis of the spectrin tandem repeat R1617 supports a model combining the ones previously postulated and suggests a much more complex scenario in which all the structural elements of spectrin contribute to the overall dynamics required for its functionality. Moreover, our analysis clearly shows that the triple helical bundles of repeats 16 and 17 require structural stabilization that is provided through intramolecular contacts by a number of central tryptophane residues^27, 48, 49^.

Taken together, HR-HDXMS might represent a powerful tool to analyze protein dynamics at single amide resolution.

## Materials and Methods

### Protein preparation

The cDNA of repeats 16 and 17 from chicken brain α-spectrin was cloned and expressed in *Escherichia coli* BL21 (DE3) (Novagen) and purified as previously described^23^.

### Sample preparation and fragmentation optimization

Conditions for optimal protein digestion were determined by adding 30 μl of quench solution with increasing concentrations of Guanidine Hydrochloride (GuHCl) (0/0.8/1.6/3.2/6.4 M GuHCl in 0.8% (v/v) formic acid) to 20 μl of sample (final concentration 0.5% (v/v) formic acid, 0/0.5/1/2/4 M GuHCl) containing 10–15 μg of protein in Tris-buffered saline (TBS: 50 mM Tris-HCl, pH 7.0 and 150 mM NaCl). Samples were then frozen on dry ice within one minute after addition of quench solution and stored at −80 °C until LCMS analysis. Sequest (Thermo Finnigan Inc) was used for peptide identification and peptides were verified using the “DXMS Explorer” software (Sierra Analytics, LLC, Modesto, CA)^19^.

### Deuterium labeling of proteins

Exchange-deuterated samples of spectrin were prepared by the addition of 15 μl of D_2_O, containing 50 mM Tris, pD 2.7 and 150 mM NaCl to 5 μl of protein stock (3 mg/ml in TBS pH 7.0) and incubated at room temperature 25 °C for 3, 10, 30, 100, 300, 10^3^, 3 × 10^3^, 10^4^, 2.5 × 10^5^ and 3.4 × 10^5^ seconds, with the reaction quenched by addition of 30 μl quench solution (0.8% (v/v) formic acid, 0.8 M GuHCl) at 0 °C, and samples immediately placed on dry ice and frozen at −80 °C until further processed (as described above). Time points of 3 and 10 seconds were incubated on ice to simulate 0.3 and 1 seconds deuteration time as there would be a 10-fold decrease in the exchange rate for every 25 °C decrease in temperature^51^. Data on the deuterated sample sets were acquired in a single automated 8 hour run, and subsequent data reduction was performed on the data reduction software (see above). Corrections for loss of D-labelling by individual fragments during HDXMS analysis (after “quench”) were made by determination of loss of deuterium from reference samples that had been fully deuterated by overnight incubation under denaturing conditions, as previously described^52–55^.

### LCMS analysis

Frozen quenched samples were placed in a Spectraphysics AS3000 autosampler kept at −45 °C with dry ice powder, rapidly thawn within 30 seconds followed by immediate injection into LC sample loop pre-cooled at 0 °C. LCMS analysis was performed at 0 °C and pH 2.3 as previously described^56, 57^. Online proteolysis involved the sample passing through an initial immobilized pepsin (30 mg/ml) column (66 μl column bed volume) that was coupled to a 20AL resin from PerSeptive Biosystems in 0.05% (w/v) trifluoroacetic acid (TFA) followed by passage through a column (66 μl column bed volume) containing immobilized protease type XIII from *Aspergillus saitoi* (20 mg/ml)^58^ at 250 μl/min flow rate. Reversed-phase separation of digested peptides utilized a C18 column, eluted by a linear acetonitrile gradient (5 to 45% B in 30 minutes; 50 μl/min; solvent A, 0.05% TFA; solvent B, 80% acetonitrile, 20% water, 0.01% TFA), with data acquisition using a Thermo Finnigan Classic LCQ electrospray ion trap type mass spectrometer or an electrospray Micromass Q-TOF mass spectrometer operated with a capillary temperature at 200 °C.

### HDXMS analysis

Mass spectrometry data was analyzed using *DXMS* data reduction software (Sierra Analytics, Version 1.01 (beta))^19^. Deuterated peptides were identified in *DXMS* and pasted into an excel macro that determines the D-incorporation of each peptide fragment at each timepoint. Protein fragmentation map, ribbon heat maps, and D-incorporation labeling curves were generated as previously described^56^. All structure figures were generated with Pymol (Delano Scientific).

### Linear least squares analysis

Given m overlapping fragments spanning residues a → b, a subfragment *x* can be defined as the overlapping and non-overlapping region(s) between fragments (Fig. S2). Given a number of subfragments *N* within a given fragment *F*, the total amount of experimentally measured deuterium ${{D}}_{{{F}}_{{\boldsymbol{n}},{\boldsymbol{t}}}}^{\exp }\,\,$ incorporated on fragment *n* at each timepoint *t* can be represented as the sum of the deuterium incorporated on each subfragment plus a residual error ${{E}}_{{{F}}_{n{,}t}}^{lin}$ as depicted in equation 1, where *j* is the defined subfragment, *N* is the total number of subfragments spanning fragment *n*, and *p* is the number of prolines that are subtracted from the number of exchangeable amides as they do not contain amide hydrogens.

1 $${{D}}_{{{F}}_{n{,}t}}^{\exp }=\sum _{{j}=1}^{{{N}}_{{n}}}{{x}}_{{j},{t}}-{p}+{{E}}_{{{F}}_{n{,}t}}^{lin}\,$$

The application of the linear program based on approximation of initial exchange rates of subfragments is illustrated in Fig. S2. The three peptide fragments *F*_*1*_, *F*_*2*_, *F*_*3*_ spanning the first 29 amino acids of spectrin define subfragments (*x*_*1*_, *…*, *x*_*5*_). The amount of D on each subfragment at each timepoint is determined by linear least square optimization to minimize the sum of the residual error over all fragments (M), and all time points (T) as defined by the global error function in equation 2.

2 $${G}{{E}}^{{\rm{lin}}}=\sum _{{t}=1}^{{T}}\sum _{{n}=1}^{{M}}{({{E}}_{{{F}}_{n{,}t}}^{lin})}^{2}$$

### Non-Linear Least Squares Analysis

The collective exchange rate *k*_*ex*_ of the amides within each subfragment is determined by simultaneously fitting the D-incorporation data to the first-order reaction kinetics for D-incorporation of an amide in equation 3, where D_*i,t*_ is the amount of D incorporated on residue *i* at time *t*.

3 $${D}_{i,t}=(1-{e}^{-{k}_{ex,i}t})$$

The rate constant ${{k}}_{{ex},{i}}$ is dependent on pH, temperature, protein sequence, and protein conformation. In a completely unstructured polypeptide chain, all peptide amide hydrogens are freely accessible to water and exchange at their maximal possible rate, with a half-life of exchange of approximately one second at 0 °C and pH 7.0 (ref. 25). Exact off-exchange rates for amide hydrogens in a fully unstructured protein sequence can be reliably calculated from knowledge of the temperature, pH and primary amino acid sequence involved^24, 25^. The precise rate of HDX of a particular amide in random coil can vary more than 30-fold from the average rates for all amides in a peptide under such conditions, with the precise rate depending upon the identity of the two amino acids flanking the particular amide bond. Because the deuterated samples are subjected to quench conditions during the LC process, a correction for back-exchange must be made to account for the loss of label from the time the sample is injected to the time it is eluted, also referred to as the lag time ${{\boldsymbol{t}}}_{{\rm{lag}}}$. We can model the back-exchange ${{D}}_{{i}}^{{bk}}$ on subfragment *x* as the fraction of remaining deuterium after a lag time of an amide at quench conditions through:

4 $${D}_{i}^{{\rm{bk}}}={e}^{-{k}_{bk,i}{t}_{{\rm{lag}}}}\,$$

where ${k}_{bk,i}$ is the calculated back-exchange rate^24, 25^ of amino acid $i$ under quench conditions at lag time ${t}_{{\rm{lag}}}$.

### Optimizing on-exchange rates by incorporating back-exchange rates

The amount of D on a single amide *i*, in a peptide fragment corrected for back-exchange can be described as the product of the amount of D-incorporated on the amide and the back-exchange according to:

5 $${D}_{i,t}^{{\rm{calc}}}=(1-{e}^{-{k}_{ex,i}t})({e}^{-{k}_{bk,i}{t}_{{\rm{lag}}}})$$

It follows that the experimental D-accumulation of a given peptide fragment F, including back-exchange corrections is the sum of the individual exchange rates of each amide plus a residual error ${E}_{{F}_{n,t}}^{{\rm{nonlin}}}$ defined by equation 6.

6 $${D}_{{F}_{n,t}}^{\exp }=\sum _{i=m}^{l}(1-{e}^{-{k}_{ex,i}t})({e}^{-{k}_{bk,i}{t}_{{\rm{lag}}}})\,+{E}_{{F}_{n,t}}^{{\rm{nonlin}}}$$

Here, ${D}_{i,t}^{{\rm{calc}}}$ is summed over all amide position *I*, *m < i < l*, where *m* and *l* are the first and *l*ast residues of fragment *n*, respectively. The *n*on-linear least squares algorithm solves for the rate constants *k*_*ex*_ that minimize the squared difference between the fitted and experimental deuteration level of all fragments. This concept is illustrated in Fig. S3. The objective function aims at minimizing the global error for all fragments ($n=1,\ldots ,M$) at all time points ($t=1,\ldots ,T$):

7 $$G{E}^{{\rm{nonlin}}}=\sum _{t=1}^{T}\sum _{n=1}^{M}{({E}_{{F}_{n,t}}^{{\rm{nonlin}}})}^{2}$$

The success of HR-HDXMS relies on the extent of overlapping fragments and the number of sampled on-exchange time points. Upper and lower boundaries are set as constraints for the fit using the intrinsic back-exchange rates determined by Molday *et al*.^24^ from spreadsheet calculations obtained at http://hx2.med.upenn.edu/download.html). The exchange rate of all prolines was set to 0 during the non-linear fit. Validation of HR-HDXMS was performed by determination of the HDX stability profile of SNase from HDXMS data and comparison with the NMR-derived stability profile. Acquisition of NMR data was performed as previously described^9^.

HR-HDXMS was written in MatLab utilizing the optimization toolbox implementing the linear and non-linear functions. The MatLab algorithm is available under http://www.hr-hdxms.us.

## Electronic supplementary material

SI Materials and Methods

Table S1
