# Supplementary material for: Computational method allowing Hydrogen-Deuterium Exchange Mass Spectrometry at single amide Resolution
Source: Sci Rep. 2017 Jun 19;7:3789. doi: 10.1038/s41598-017-03922-3 (PMC5476592; doi:10.1038/s41598-017-03922-3)
Supplement: Supplementary file 1 — SI Materials and Methods [file 41598_2017_3922_MOESM1_ESM.pdf]

Computational method allowing Hydrogen Deuterium Exchange Mass Spectrometry at single amide Resolution

Chris Gessner[1], Wieland Steinchen[5], Sabrina Bédard[3], John Skinner[4], Virgil L. Woods, Jr[6]., Thomas J. Walsh[2], Gert Bange[5] and Dionysios P. Pantazatos[2, *]

[1]Indiana University, Department of Informatics and Computing, Bloomington, IN, USA

[2]Weill Cornell Medicine, Transplantation-Oncology Infectious Disease Program, Division of Infectious Diseases 1300 York Ave New York, NY 10065

[3] GlaxoSmithKline, Platform Technology & Science, Collegeville Road, Collegeville, Pennsylvania 19426 United States

[4] iHuman Institute, ShanghaiTech University, 99 Haike Road, Pudong, Shanghai, China

[5] Philipps-University-Marburg, Faculty of Chemistry & LOEWE Center for Synthetic Microbiology

Hans-Meerwein-Strasse, C7 35043 Marburg, Germany

[6]deceased

* Correspondence: dpantaza@gmail.com Tel: +1 646-697-0044

The SI Material and Methods includes:

Supplementary Figures 1 – 6

Supplementary Table 1

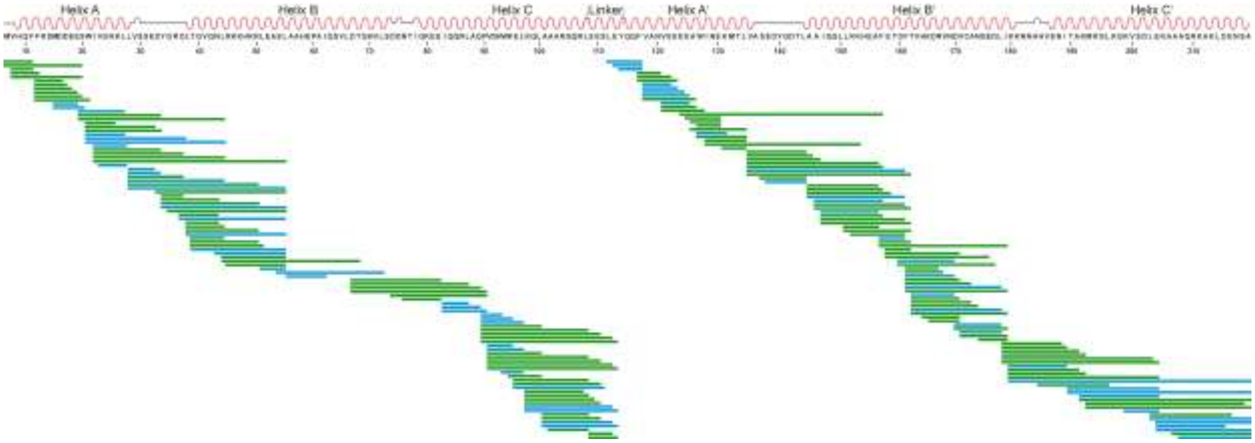

Supplementary Figure 1. Peptide coverage map of α-spectrin. Peptides obtained by digestion of α-spectrin with pepsin or pepsin plus fungal protease XIII are shown in blue and green, respectively. The secondary structure is depicted above the amino acid sequence. Residues are labelled according to their position in PDB: 1CUN[1].

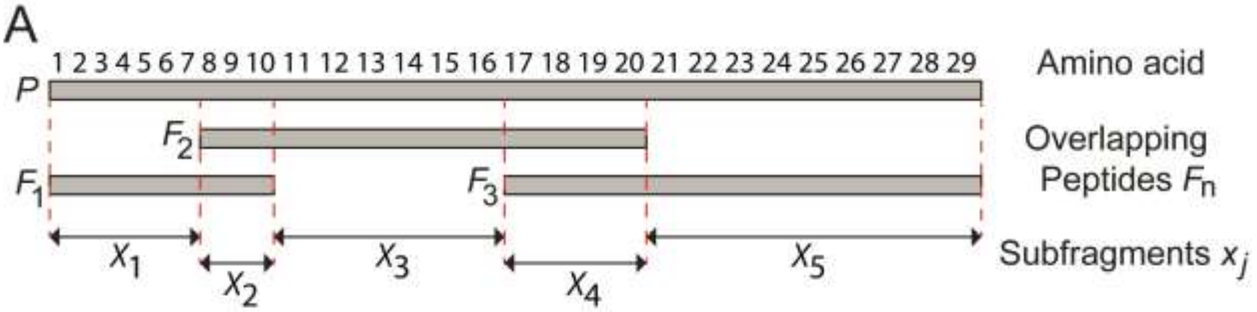

B

Equation 1 $$D^{exp}_{F_{n,t}} = \sum_{j=1}^{N_n} x_{j,t} - p + E^{lin}_{F_{n,t}}$$

Equation 2 $$GE^{lin} = \sum_{t=1}^{T} \sum_{n=1}^{M} \left(E^{lin}_{F_{n,t}}\right)^2$$

Peptide (aa)	#D (10s)	#D (30s)
F_1 (1-10)	3.75	4.29
F_2 (8-20)	5.62	7.23
F_3 (17-29)	2.34	2.45

Subject to:

	#D (10s)	#D (30s)
F_1	$x_{1,10} + x_{2,10} + E_{1,10} = 3.75$	$x_{1,30} + x_{2,30} + E_{1,30} = 4.29$
F_2	$x_{2,10} + x_{3,10} + x_{4,10} + E_{2,10} = 5.62$	$x_{2,30} + x_{3,30} + x_{4,30} + E_{2,30} = 7.23$
F_3	$x_{4,10} + x_{5,10} + E_{3,10} = 2.34$	$x_{4,30} + x_{5,30} + E_{3,30} = 2.45$

Supplementary Figure 2. Example of linear least squares analysis in HR-HDXMS. A. The overlapping peptides F_1 (aa 1-10), F_2 (aa 8-20) and F_3 (aa 17-29) produce the five overlapping subfragments X_1-X_5. **B.** The linear least squares analysis aims at minimizing the error $E^{\text{lin}}_{F_{n,t}}$ of deuterium incorporation for each peptide F composed of the subfragments X_j at a given time of deuteration. The inset depicts exemplary values for the amount of deuterium incorporated by each of the peptides F_1, F_2 and F_3 after 10 and 30 seconds of deuteration. The experimental deuterium incorporation $D^{\text{exp}}_{F_{n,t}}$ of a peptide F is the sum of deuterons of all subfragments x_j plus a residual error $E^{\text{lin}}_{F_{n,t}}$ (equation 1). The linear global error (GE^{lin}) function (equation 2) is the sum of the squared residuals for all fragments ($n = 1, \ldots, M$) at all time points ($t = 1, \ldots, T$).

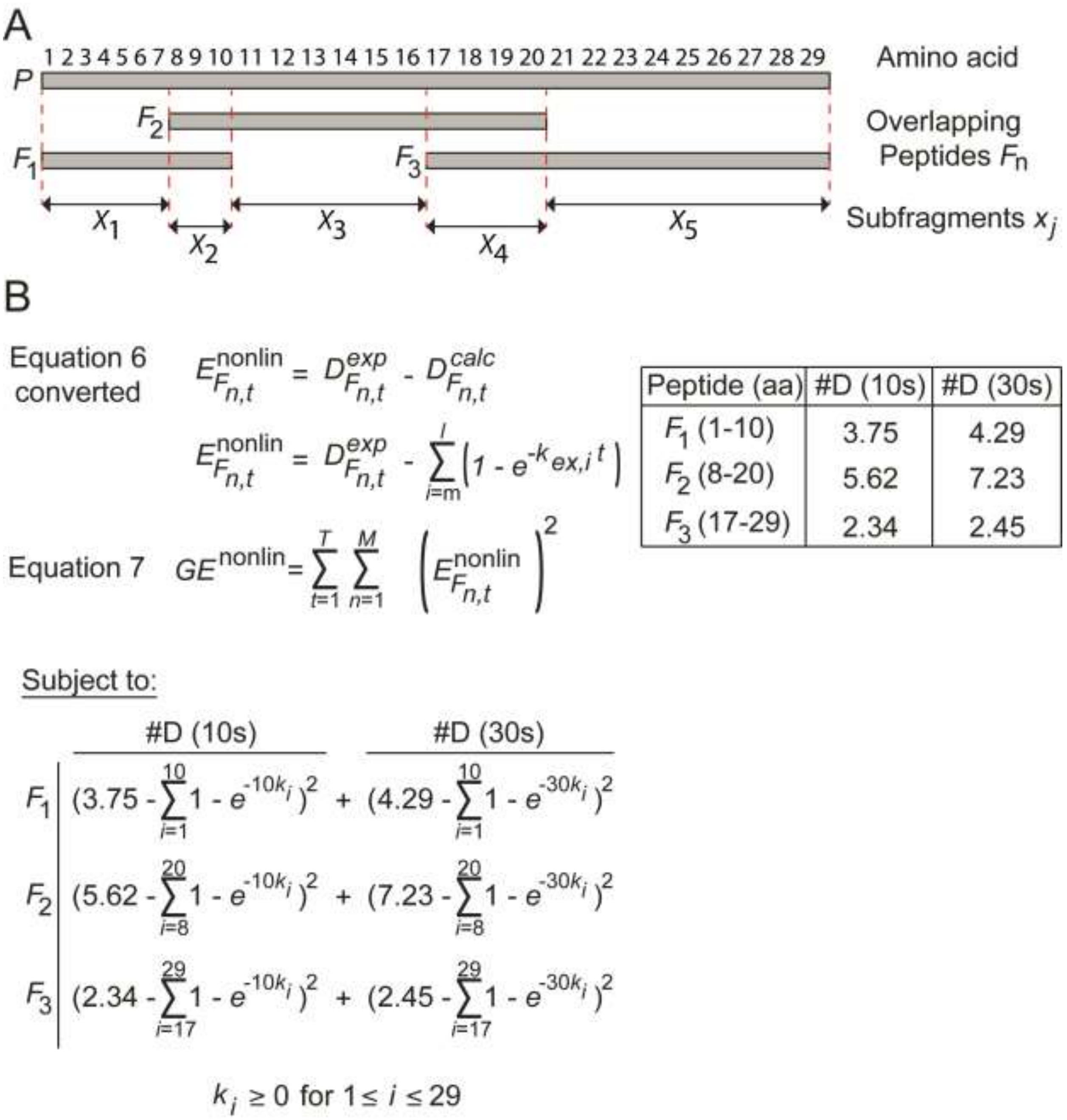

Supplementary Figure 3. Example of non-linear least squares analysis in HR-HDXMS. A. The overlapping peptides F_1 (aa 1-10), F_2 (aa 8-20) and F_3 (aa 17-29) produce the five overlapping subfragments X_1-X_5. **B.** The non-linear least squares algorithm solves for the rate constants $k_{ex,i}$ that minimize the squared difference (*i.e.* $E^{\text{nonlin}}_{F_{n,t}}$) between the fitted ($D^{\text{calc}}_{F_{n,t}}$) and experimental ($D^{\text{exp}}_{F_{n,t}}$) deuteration level of all fragments (equations 6 and 7). The inset depicts exemplary values for the amount of

deuterium incorporated by each of the peptides F_1, F_2 and F_3 after 10 and 30 seconds of deuteration. For reasons of simplicity, the example calculation only depicts the on-exchange rates $k_{ex,I}$ without correction for back-exchange rates $k_{bk,I}$ (equation 6). Further details are given in the text.

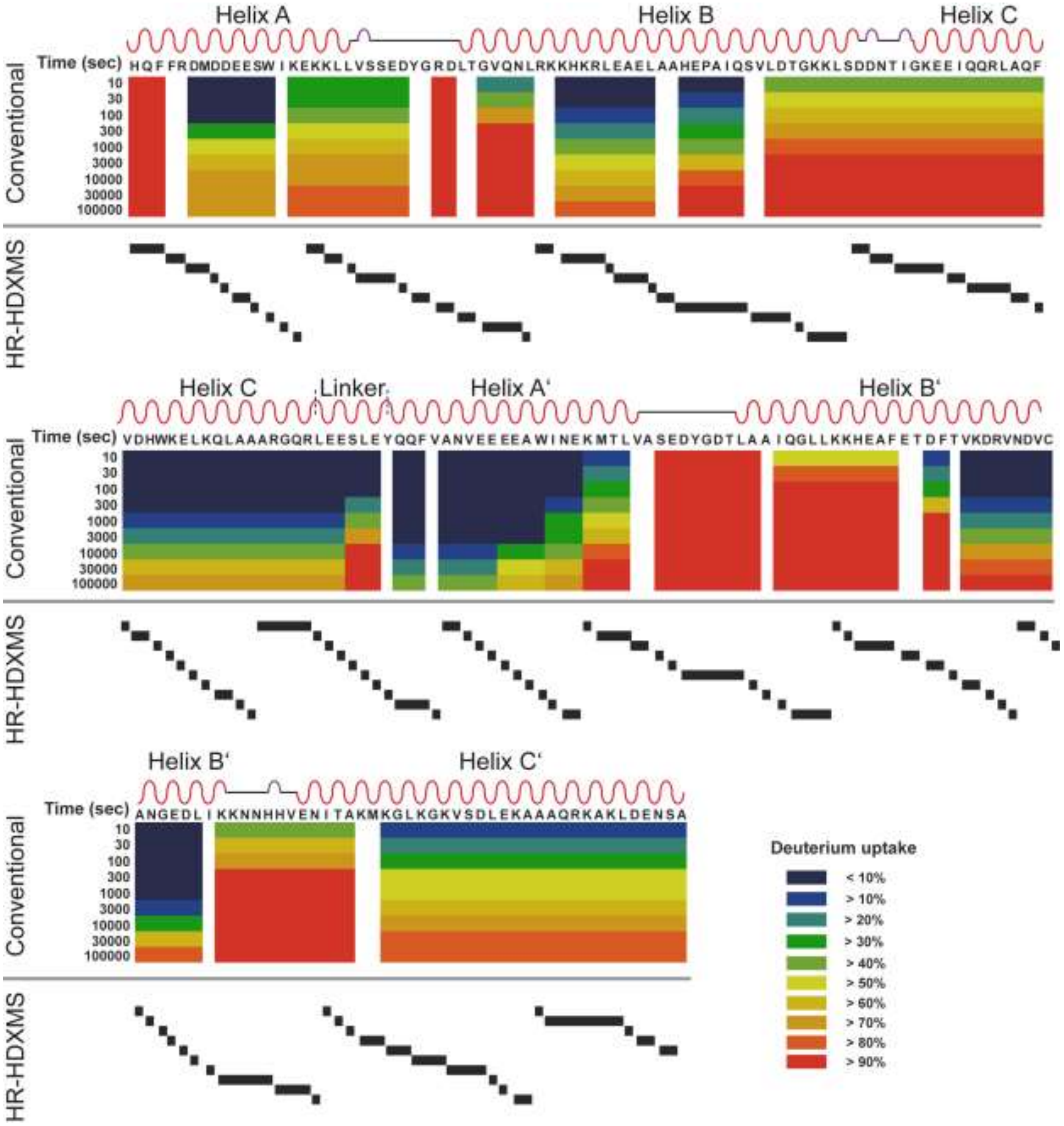

Supplementary Figure 4. Improved resolution of α-spectrin conformational dynamics by HR-HDXMS. *Upper panel:* Ribbon map of selected peptides of α-spectrin colored according to their percentage deuterium uptake represents the 'conventional' way of HDX data analysis and visualization. *Lower panel*: HR-HDXMS improves the resolution of HDX data analysis. Each black bar indicates a subfragment generated by HR-HDXMS.

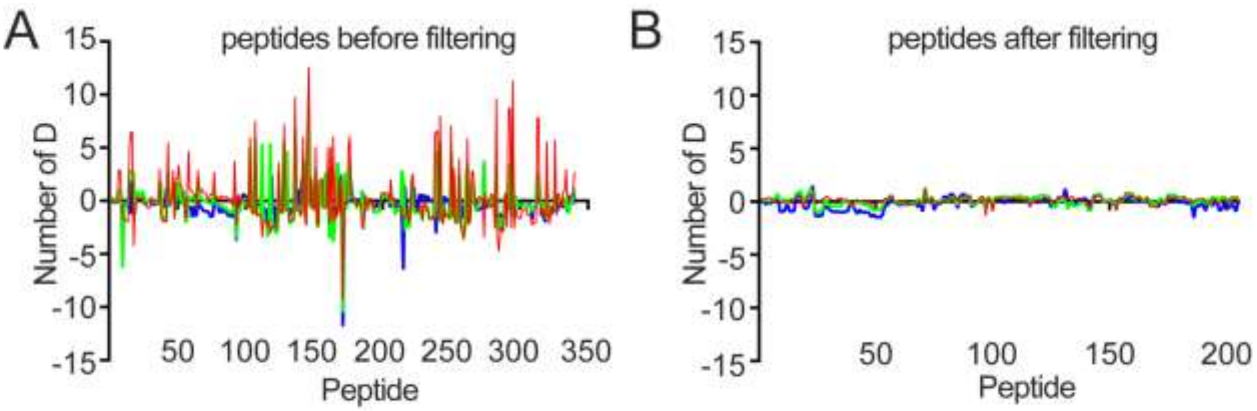

Supplementary Figure 5. Quality of HDXMS data significantly affects residual error in HR-HDXMS. A. A large number of residues exhibited a residual error during linear least squares analysis of SNase (equation 1). **B.** Elimination of peptides with an error ≥ 2 deuterium (D) reduces the total global error of the linear optimization (equation 2). **A-B.** Deuterium incorporation for each peptide is shown after 60 (red), $6x10^3$ (green) or $5.2x10^5$ (blue) seconds of deuteration.

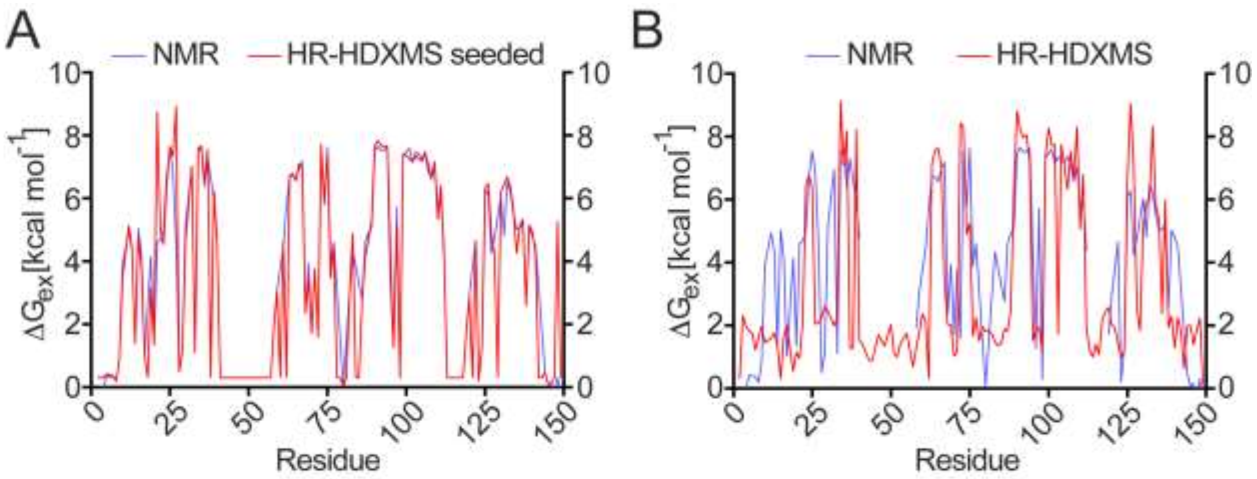

Supplementary Figure 6. Validation of protein stability fingerprint of staph nuclease. A. Overlay of the stability fingerprints of SNase generated from the k_{ex} values obtained by NMR[2] (blue line) or by applying the k_{ex} values from NMR to the HR-HDXMS algorithm (red line, equations 1 and 6). **B.** Overlay of the stability fingerprints of SNase generated from the k_{ex} values obtained by NMR[2] (blue line) or from the k_{ex} values determined from the HDX dataset of staph nuclease through the linear and nonlinear least squares analysis by the HR-HDXMS algorithm (red line, equations 1 and 6, compare to **Figs. S2** and **S3**).

SUPPLEMENTARY REFERENCES

1. Grum, V. L., Li, D., MacDonald, R. I. & Mondragon, A. Structures of two repeats of spectrin suggest models of flexibility. *Cell* **98**, 523-535 (1999).
2. Skinner, J. J., Lim, W. K., Bedard, S., Black, B. E. & Englander, S. W. Protein hydrogen exchange: testing current models. *Protein Sci* **21**, 987-995, doi:10.1002/pro.2082 (2012).
